# A Refined Methodology for Defining Plant Communities Using Postagricultural Data from the Neotropics

**DOI:** 10.1100/2012/365409

**Published:** 2012-03-12

**Authors:** Randall W. Myster

**Affiliations:** Biology Department, Oklahoma State University, Oklahoma City, OK 73107, USA

## Abstract

How best to define and quantify plant communities was investigated using long-term plot data sampled from a recovering pasture in Puerto Rico and abandoned sugarcane and banana plantations in Ecuador. Significant positive associations between pairs of old field species were first computed and then clustered together into larger and larger species groups. I found that (1) no pasture or plantation had more than 5% of the possible significant positive associations, (2) clustering metrics showed groups of species participating in similar clusters among the five pasture/plantations over a gradient of decreasing association strength, and (3) there was evidence for repeatable communities—especially after banana cultivation—suggesting that past crops not only persist after abandonment but also form significant associations with invading plants. I then showed how the clustering hierarchy could be used to decide if any two pasture/plantation plots were in the same community, that is, to define old field communities. Finally, I suggested a similar procedure could be used for any plant community where the mechanisms and tolerances of species form the “cohesion” that produces clustering, making plant communities different than random assemblages of species.

## 1. Introduction

The study of plant communities has been problematic, in part not only because of the various ways available to define them, but also due to the continuing debate about whether they even exist [1, 2]. Opinion has varied from a belief in strongly interacting plant communities [3], to the more commonly accepted individualistic view of plant assemblages [4]. But even if species behave individualistically, they may still form communities due to, for example, similar responses to mechanisms and overlapping tolerances [5]. These issues continue to influence how plant ecologists think of such constructs as biomes, ecotones, and ecoclines [6].

 Criteria for a plant community may be that it only has a nonrandom subset of the regional pool of available species [7]. Alternatively, a plant community may contain properties such as (1) assembly rules that filter out species and traits until a community is left with only the most well-adapted species [8], (2) niche limitation, (3) stability, (4) resilience, (5) discontinuity/discreteness, (6) self-organization, (7) emergence, (8) coevolution [1, 9–11], or (9) “integratedness” such as linkage between processes [12]. Indeed, different communities may be (1) areas with different physiognomies [2, 9], (2) areas that contain species with different C, S, or R affinities [13], or (3) areas that have different functional groups. Plant communities may even be made up simply of complementary guilds of plants that share resources, such as light, water, and soil nutrients [14].

 A common approach has been to first find broad structural characteristics that all plant communities must have—such as species composition, species richness, species evenness, and biomass [14]—and then measure those characteristics in field plots. An “index” based on these characteristics may also be computed [15]. If the variation of selected traits, or said index, within a subset of plots is small compared to the variation among all plots then those plots are considered to be in the same community [16]. This methodology is implicit in multivariate ordinations which group vegetation quadrats into community types according to how far apart they are in an ordination “space” defined on axes that are correlated with specific plant species, soil factors, or other parameters measured in those quadrats [17]. The problem with this approach [18–20] is that it does not necessarily include whether or not the plant species common to the plots actually occur together over the larger spatial and temporal scales of their distributions, where they associate naturally, which is fundamental to what makes a plant community.

 In this paper I (1) start with the observation that positive plant associations among species are central to defining plant communities regardless of the mechanisms and/or tolerances that produce them, (2) suggest that the key question of whether or not two plots are in the same plant community is not answerable as “yes or no” but only in terms of degree, and hence (3) compare recently sampled plots to a hierarchy, built from positive plant associations taken from many plots sampled over time from the same community type [21]. Such long-term plots are needed to observe the natural “affinity” that these plant species have for each other.

 Here I show how to define a common plant community (old fields) using postagricultural data sampled in the Neotropics by first computing all significant pairwise plant species associations in plots from five abandoned pastures and plantations sampled annually for a decade and then clustering those associations into a hierarchy using an association metric of decreasing strength [22, 23]. Finally I show how the key question of whether or not two plots are in the same community can be answered using that hierarchy. Such an approach can thus be used to define any plant community because it contains degrees of integration [11] and also captures the individualistic, overlapping distributions of plants found over space on gradients [24] and over time after a disturbance [25].

## 2. Methods and Materials

All five study pastures and plantations are located within tropical lower montane wet forests [26] of similar plant taxa [21]. All study areas receive between 3 m and 5 m of rain annually with small seasonally variation [27], and their temperatures range between 15°C and 25°C. All soils are fertile andisols and volcaniclastic in origin [28, 29]. The study pasture was never seeded with grasses and grazed for decades before abandonment. The pasture borders the Luquillo Experimental Forest (LEF) of northeastern Puerto Rico USA, close to the town of Sabana (18°20′N, 65°45′W: [30–34]) where the LEF is a long-term ecological research (LTER) site of the National Science Foundation (http://luq.lternet.edu/). The two study banana (*Musa *sp.) plantations (named left and right for convenience) and the two study sugarcane (*Saccharum officinarum*) plantations (also left and right) are located in the Maquipucuna Reserve, Ecuador (0°05′N,78°37′W; http://www.maqui.org; [35–40]).

 Within each study pasture and plantation, twenty-five 5 m × 2 m contiguous plots were laid out in 1996 [33] with the long side parallel to and bordering the forest in order to maximize any edge effects. Past analysis of this plot data [21, 32, 39] has shown them to be of sufficient size to capture community structure. No plots had any remnant trees or sprouting tree roots at the beginning of the study, and their tree seed bank was very small [34]. Starting in May of 1997, and continuing annually in May since then, each plot has been sampled for percent cover of each plant species. Percent cover—an indication of a species' ability to capture light and, therefore, to dominate these areas which are in the process of becoming forested communities [32]—was estimated visually in relation to each plot's area. Trained on-site LTER plant taxonomists were employed to identify plant species in Puerto Rico and plant taxonomists, trained at the University of Georgia where voucher specimens are kept on file [41, 42], assisted in the identification of species by using specimens located on site in Ecuador [30, 38]. The data from the plots in Puerto Rico (LTERDATB no. 97) and Ecuador (LTERDATB no. 101) are housed in the archives of the LEF LTER site.

 First quantitative percent cover data, not presence/absence data, were used to generate pairwise Spearman coefficients of rank association [21, 26, 43, 44]. For each sampling year and field, the percent cover of any two species in each of the 25 plots (containing very few zeros) was used to compute a pair-wise association coefficient. Only the statistically significant (alpha < 0.05) positive associations are reported here but all associations, both negative and positive, can be found in [21]. Because only the first ten years of sampling data were used for each pasture and field, there is a maximum of 10 significant positive associations possible between any two plant species in Tables 1–5. This matrix of associations were then used to generate dendrograms for each separate pasture and field, after subtracting each cell value from a possible maximum of 10, using Ward agglomerative clustering [19, 43, 45] shown best for ecological data [46]. Clusters begin as single species and then form association clusters of more and more species (a hierarchy using species cooccurrence over large areas: [47]) based on a metric that becomes weaker as species form groups, eventually leading to all species clustered in one large group. Finally it should be remembered that any results given here may hold only for the original plot size.

## 3. Results

All pastures and fields showed a low amount of positive association in the context of the 6760 positive associations possible given all 26 species over 10 years. While no pasture/field showed more than 5% of the possible total (Table 1), the pasture in Puerto Rico had the greatest number of associations. In the Puerto Rican pasture, species that formed many positive associations included the trees *Syzygium, jambos, Guarea guidonia*, *Ocotea leucoxylon*, and *Prestoea montana* (Table 2) and for the left banana plantation of Ecuador key species with many positive associations included *Begonia *spp., *Trichipterix pilosissima*, and *Ochroma *spp. (Table 3). In the right banana plantation, *Begonia *spp., *Cuphea *spp., and *Brugmansia *spp. formed many associations (Table 4). In the left sugarcane plantation, key species included members of the families Asteraceae, Verbenaceae, and Papilionacea (Table 5), and in the other sugarcane plantation, *Cuphea *spp. and *Piper aduncum *were important (Table 6).

**Figure 1:**
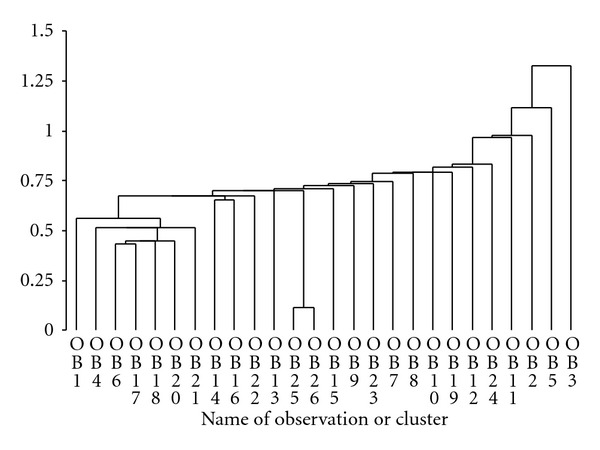
Dendrogram for pasture species (OB1 , OB2 , … , OB26) which are numbered as in Table 2.

**Figure 2:**
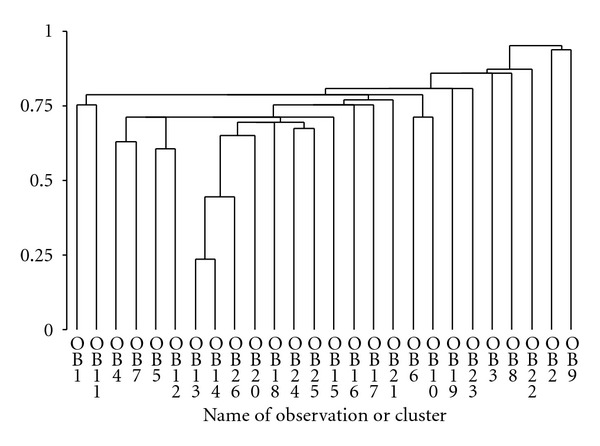
Dendrogram for left banana species (OB1 , OB2 , … , OB26) which are numbered as in Table 3.

 Clustering of the data in Table 2 (Puerto Rican pasture) showed that *Myrcia* and *Ocotea *clustered first, followed at a longer metric by *Desmodium* and *Piper*, which quickly formed a cluster with *Andira* and *Miconia*. That cluster fused then with *Citrus* and *Psychotria *and then with *Bromelia*. Then the rest of the species fused with all the previously mentioned species and clusters, except for *Prestoea*, *Syzygium* and *Ocotea* (Figure 1). Clustering of the data in Table 3 (left banana) showed that *Setaria* and *Bocconia* clustered first, united with *Passiflora* next, which clustered with *Alternanthera* sp. at about the same level as clusters form between *Musa *and *Anthurium* and between *Cyathea* and other *Anthurium*. The other species then clustered quickly, with *Begonia* and *Nectandra* forming a cluster last (Figure 2).

**Figure 3:**
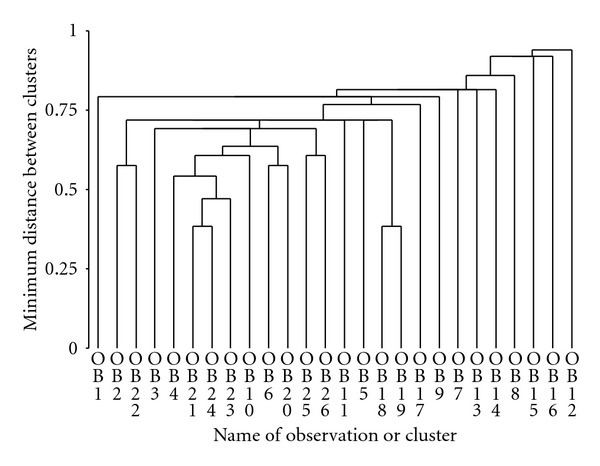
Dendrogram for right banana species (OB1 , OB2 , … , OB26) which are numbered as in Table 4.

**Figure 4:**
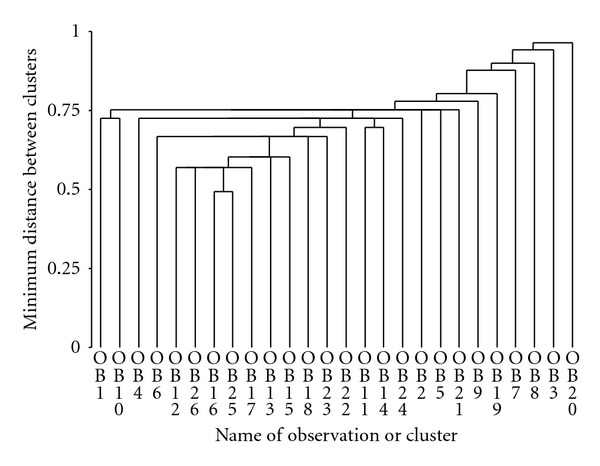
Dendrogram for right sugarcane species (OB1 , OB2 , … , OB26) which are numbered as in Table 5.

**Figure 5:**
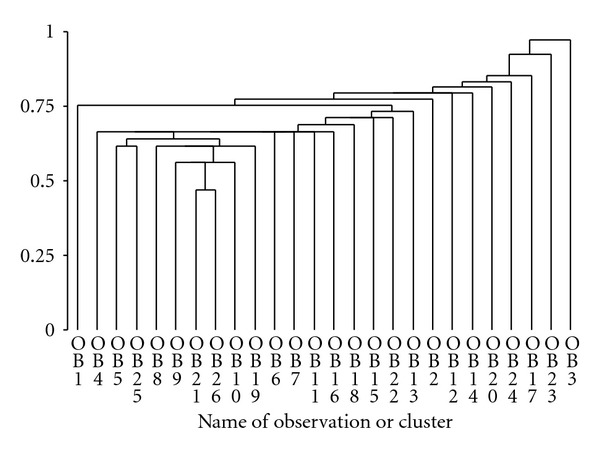
Dendrogram for left sugarcane species (OB1 , OB2 , … , OB26) which are numbered as in Table 6.

Clustering of the data in Table 4 (right banana) showed that *Inga* and *Crataegus* form a cluster at the same level as *Commelina* and *Erythrina*. The *Inga* cluster then fused with *Chenopodium* and later *Musa*. After that there were three clusters that formed between two species each: *Costus*/*Musa*, *Setaria*/*Heliotropium,* and *Bocconia*/*Cecropia*. A large cluster then formed which included all of the previously mentioned species plus *Vernonia* and *Piperaceae*. The rest of the species were added with *Chusquea, Nectandra,* and *Begonia* clustering last (Figure 3). Clustering of the data in Table 5 (right sugarcane) showed that *Costus* and *Columnea *formed the first cluster and it then united with *Rubus*, Orchidaceae, and *Miconia*. This cluster then united with *Commelina* and *Cecropia, *making a larger cluster that then joined with *Passifloraceae* and *Hieracium*. After this clustering, levels were similar among species until the end when *Piper*, *Lantana*, *Digitaria,* and *Chusquea* clustered last (Figure 4). Finally, clustering of the data in Table 6 (left sugarcane) showed that *Nectandra* and *Polypodiaceae* clustered first, then with *Asteraceae* and *Baccharis*, followed by *Sida* and *Commelina*. At the same level *Miconia* and *Vernonia* cluster and all of these species then join to be added with *Piper*, *Rubus*, *Polpyodiaceae,* and *Saccharum*. Finally the last species to cluster were *Piper*, *Orchidaceae,* and *Cuphea* (Figure 5).

## 4. Discussion

Because most of the significant associations in the study plot data were positive—unlike the mainly negative associations that were computed from plots in temperate old fields (using the same plot grid layout, sampling protocol, and analysis [26])—facilitation in these stressful, early successional fields may be more important than competition (also see [48]) which would challenge ecological paradigms regarding the pervasiveness of competition [49, 50].

 The Puerto Rican pasture is different from the other fields with both different species and a different clustering pattern, although *Miconia *does cluster early here and in both sugarcane fields. Unfortunately without replication the cause of this difference—for example, it is because it is a pasture, because it revegetated naturally rather than was seeded with grass, because it is an island, or because it is Puerto Rico—cannot be determined. However both replicate banana fields in Ecuador show (1) *Musa* (their past crop) clustering in the middle of the pack of species and (2) that *Begonia* and *Nectandra* are the last two species to cluster. This suggests that recovering banana fields have distinct communities. In the sugarcane fields, (1) the past crop *Saccharum* is not as persistent as *Musa* was in the banana fields, (2) *Miconia* and *Commelina* clustered in both fields but at different levels, (3) *Acalypha* and *Erythrina *clustered in the middle of the pack, and (4) *Piper* clustered last in both fields. Consequently evidence for repeatable communities occurs in recovering sugarcane and banana plantations, but it is stronger in the banana plantations. In general, species groupings do not suggest taxonomic or obvious ecological (e.g., dispersal vector, seed size, shade tolerance) similarities, but there is a suggestion that the past crop not only persists after abandonment but also forms associations with invading plants [26].

 Results suggest that whether two plots are in the same plant community is not a “yes/no” proposition but rather a level of a hierarchy derived from the significant positive associations among the constituent plants themselves. This reflects the known individualistic, overlapping plant distribution patterns over both space (on gradients: [25]) and time (after a disturbance: [51]) where tolerances and mechanisms [21] produce the “cohesion” that makes plant communities something different than random assemblages of species.

 How then should someone decide if two plots are in the same plant community, that is, how should we define a plant community? I suggest first deciding which plot species to focus on in the association analysis and then consulting the association hierarchies derived from long-term, repeated sampling of the same kind of communities (here, postagricultural) to find the level of association needed to cluster those species together. Defining what level defines a community is, of course, a basic issue in clustering methodologies [19] where taking all species as defining their own individual communities, or defining only one community which contains all species, is not ecologically meaningful. One possible way to choose this level of association intensity could be based on the ecology and biology of the species themselves and/or the ecosystem they are found in. Alternatively, one may look for “cleavage points” in the clustering pattern where the break between groups is most clear, thereby making communities most distinct from each other. Or one may simply say that the association clustering pattern “is” the plant community. Finally it should be noted that association hierarchies may define communities that do not currently exist but may exist, or could have existed, at some other place and time.

 In this paper I began with the observation that positive plant associations among species are central to defining plant communities regardless of the mechanisms and/or tolerances that produce them. I then suggested that the key question of whether or not two plots are in the same plant community is not answerable with “yes or no” but only in terms of degree. This leads to the construction of a hierarchy, built from positive plant associations after decades of plot sampling in the same kind of community (old fields) using many plots located in different areas and using an association metric of decreasing strength. Only then can recently sampled plots be compared to the hierarchy to decide whether or not they are in the same community.

 The association hierarchy and its clustering metric can be interpreted as showing (1) assembly rules defining a colonization process of permissible or forbidden species combinations [52], (2) functional groups where positive association means that species respond similarly to environmental factors and gradients [26], or (3) intrinsic “guilds” built up from community data [10]. Using this postagricultural data set we may also be able to address whether similar species group together regardless of whether they are in pasture, banana, or sugarcane (i.e., community convergence or divergence: [10]). Finally I suggest that future community investigations follow the sampling protocol of this data set with a hierarchy containing enough plots and a long enough sampling time to allow for significant individual plant-plant associations to develop as plants replace each other over time [21, 53]. Such an approach makes it much more likely that the species groupings that define actual plant communities will be found.

## Figures and Tables

**Table 1:** Positive association summary.

Country/field type	Total no. of positive associations	Largest no. for any year and plot
Puerto Rico USA/pasture	342	7
Ecuador/left banana	159	4
Ecuador/right banana	133	4
Ecuador/right sugarcane	132	4
Ecuador/left sugarcane	158	4

**Table 2:** Half-matrix containing the number of significant positive spearman rank correlation coefficients among all plant species in a Puerto Rican pasture for each year over the first 10 years of succession, with a maximum of ten. Plant species and families are (1) *Bromelia* spp., (2) *Guarea guidonia*, (3) *Ocotea leucoxylon*, (4) *Citrus frutius*, (5) *Syzygium jambos*, (6) *Desmodium* spp., (7) *Gleichenia bifida*, (8) *Inga laurina*, (9) *Citrus limon*, (10) *Casearia sylvestris*, (11) *Prestoea montana*, (12) *Calophyllum calaba*, (13) *Miconia prasina*, (14) *Eugenia pseudopsidium*, (15) *Tabebuia heterophylla*, (16) *Eugenia malaccensis*, (17) *Piper hispidum*, (18) *Andira inermis*, (19) *Psychotria brachiata*, (20) *Miconia racemosa*, (21) *Psychotria berteriana*, (22) *Xanthosoma* spp., (23) *Clidemia hirta*, (24) *Panicum* spp., (25) *Myrcia splendens,* and (26) *Ocotea sintenisii*.

	2	3	4	5	6	7	8	9	10	11	12	13	14	15	16	17	18	19	20	21	22	23	24	25	26
1	1	1		2		1		2		1	3			2			1								2
2		6	2	7	2	3			2	5	2	1		3				1	1	3	1	2	2	4	2
3			1	3		1	1	2	4	2	3	1	3	2	3							2	4	4	2
4				1	2	2			1	1	1		1	1		2	1		1						
5					1	1	4	2		5	7	2	1	2	2		1		2	2	3	1	4	3	2
6						2		1		1							1					1	1		
7							1	2	2	1	4				1	1		3		1		1	2		1
8										5		1	3		4			1		1	1	1			2
9										2	1	1		2	1	3	1	1	1			1	1	1	5
10											2		2	2	1			5					2	2	2
11														2	2	2	1	1	1	2		3	1	2	3
12												2	1	1	2	1			3				2	3	1
13														4	2		1	4	1			2	1		
14														2	1					1		2	2		
15																			1		1				1
16																			1			2	1	3	1
17																									
18																						2			
19																				1		1			
20																					1	4		2	1
21																									
22																							2	2	
23																									1
24																								4	1
25																									1
26																									

**Table 3:** Half-matrix containing the number of significant positive spearman rank correlation coefficients among all plant species in the Ecuador left banana plantation for each year over the first 10 years of succession. Plant species and families are (1) *Acalypha plalycephatus*, (2) *Begonia* spp., (3) *Geonoma undata*, (4) *Cyathea* spp., (5) *Musa* spp., (6) *Pilea* spp., (7) *Anthurium* spp., (8) *Trichipterix pilosissima*, (9) *Nectandra* spp., (10) *Ochroma* spp., (11) *Baccharis* spp., (12) *Anthurium* spp., (13) *Setaria* spp., (14) *Bocconia frutescens*, (15) *Piper aduncum*, (16) *Erythrina megistophyllta*, (17) *Vernonia patens*, (18) *Hedyosmum* spp., (19) *Commelina diffusa*, (20) *Alternantcera* spp., (21) *Siparuna piloso-lepidota*, (22) *Solanum* spp., (23) *Vernonia* spp., (24) *Digitaria sanguinalis*, (25) *Inga* spp., and (26) *Passiflora* spp.

	2	3	4	5	6	7	8	9	10	11	12	13	14	15	16	17	18	19	20	21	22	23	24	25	26
1	1				1	1	2		1	1		2	2						2	1					2
2		2	2	2	4	1	1		2	1	2									1	1	1			
3			3	1	1	3	1	3			1														
4					1	1	1	3						1	1										
5					2	1								1			1								1
6							2	1		2	1								1			1			
7							3	3		1												1			
8								3	2	1									1		1	2		1	
9									2											1					
10										3	2								1	1	1	1	1	1	1
11												2	2	2				1					1		1
12												1					1		1	1	1		1		
13														1											1
14																									1
15															3	1									
16																2							1		
17																	2	2						2	
18																		3	1						
19																			2		1				
20																				1	1				
21																					5				
22																							2		
23																							2	3	
24																								1	1
25																									
26																									

**Table 4:** Half-matrix containing the number of significant positive spearman rank correlation coefficients among all plant species in the Ecuador right banana plantation for each year over the first 10 years of succession. Plant species and families are (1) *Acalypha plalycephaluss*, (2) *Costus* spp., (3) *Musa* spp., (4) *Solanum muricatum*, (5) Piperaceae, (6) *Setaria* spp., (7) *Tagetes terniflora*, (8) *Begonia* spp., (9) *Cuphea carthlagenensis*, (10) Polypodiaceae, (11) *Vernonia patens*, (12) *Brugmansia* spp., (13) *Digitaria sanguinalis*, (14) Urticaceae, (15) *Chusquea* spp., (16) *Nectandra* spp., (17) Piperaceae, (18) *Commelina diffusa*, (19) *Erythrina megistophyllta*, (20) *Heliotropium* spp., (21)* Inga* spp., (22) *Musa acuminate*, (23) *Chenopodium album*, (24) *Crataegus monogyna*, (25) *Bocconia frutescens,* and (26) *Cecropia monostachyta*.

	2	3	4	5	6	7	8	9	10	11	12	13	14	15	16	17	18	19	20	21	22	23	24	25	26
1		1				1	3	1	1			1			1	1									
2		1	1	1	2			2	1										1						
3						1		2			2	1	1	1						1					1
4									1	1			1	1		1								2	1
5						3			1	1	1				1						1				
6																	1	1			2				
7							4	2	1	1							1								
8								2	1	1				1	2	1									
9									1	1	1	1				1				1	2		1		
10										1		1		1										2	1
11											3							1							
12												2	1		1	1	1	1	1	1					1
13													3	3					1		1				
14														3										1	1
15																3									
16																	3	3							
17																			2	1				1	1
18																			1						
19																					1				
20																					3				
21																								1	2
22																									
23																								1	
24																									2
25																									
26																									

**Table 5:** Half-matrix containing the number of significant positive spearman rank correlation coefficients among all plant species in the Ecuador right sugarcane plantation for each year over the first 10 years of succession. Plant species and families are (1) *Acalypha pladichephalus*, (2) Asteraceae, (3) *Digitaria sanguinalis*, (4) Polypodiaceae, (5) *Nectandra* spp., (6) *Stachys micheliana*, (7) Piperaceae, (8) *Lantana camara*, (9) Verbenaceae, (10) *Erythrina megistophyllta*, (11) *Piper aduncum*, (12) *Rubus* spp., (13) *Commelina diffusa*, (14) *Elephantopus mollis*, (15) *Cecropia *spp., (16) *Costus* spp., (17) *Miconia *spp., (18) Passifloraceae, (19) Fabaceae, (20) *Chusquea* spp., (21) Marantaceae, (22) *Pilea* spp., (23) *Hieracium* spp., (24) *Sabicea* spp., (25) *Columnea* spp., and (26) Orchidaceae.

	2	3	4	5	6	7	8	9	10	11	12	13	14	15	16	17	18	19	20	21	22	23	24	25	26
1	1	1	1	2			2			1	1									1	1		1		
2			1	1	1	4		2														1	2	1	
3														1							2	2	3		
4				1				1		1			2	2		1			1			1			
5					1	2		2																	1
6						1		2					1				1	1	1				1		
7								1		1							1					1			
8								1	3									1	1	2	2				
9											1		2	1						1			1		
10										1	1		2							1	1				
11											2						1						1		1
12													2									1		1	
13														1	1				3	1					
14														1									1	1	1
15																			1				1		
16																		1	1						
17																	3							1	
18																		2	1						1
19																			2	2	1	1	1	2	
20																				3	1			1	
21																					1	1			
22																									
23																							1		
24																									
25																									1
26																									

**Table 6:** Half-matrix containing the number of significant positive spearman rank correlation coefficients among all plant species in the Ecuador left sugarcane plantation for each year over the first 10 years of succession. Plant species and families are: (1) *Musa* spp., (2) *Costus* spp., (3) *Cuphea carthagenensis*, (4) *Digitaria sanguinalis*, (5) *Miconia* spp., (6) *Piper* spp., (7) *Rubus* spp., (8) *Sida rhombifolia*, (9) Asteraceae, (10) *Baccharis* spp., (11) Polypodiaceae, (12) *Lantana camara*, (13) *Vernonia patens*, (14) *Acalypha pladichephalus*, (15) *Solanum* spp., (16) *Saccharum officinarum*, (17) *Piper aduncum*, (18) Verbenaceae, (19) *Commelina diffusa*, (20) *Erythrina megistophyllta*, (21) *Nectandra* spp., (22) *Altus* spp., (23) Orchidaceae, (24) *Polybotrya* spp., (25) *Vernonia* spp., and (26) Polypodiaceae.

	2	3	4	5	6	7	8	9	10	11	12	13	14	15	16	17	18	19	20	21	22	23	24	25	26
1		1				1			1		1			1	2	3	1		1				1		1
2		2	2	2											1			1			1	3	1	1	
3			3	1	4	2	1			3											1			1	
4				1	3	2	1	1			1														
5					1	1	1				1											1	1		
6							1			1			1				1						1		
7							1		2	1							1	1	1				1	1	
8										1			1		1		1						1	1	
9												1			2					1		1	1	1	1
10											1	1		1							1				
11																1					1				
12													4	1				1				1	1	1	
13													1	2				2		1					2
14														2		1	1	1				2	1		
15															1	2		2	1			1			
16																1	2					1			
17																	1					4		1	
18																		2			1			1	
19																					1	1	1		
20																					4	2		1	2
21																						1			
22																									
23																							3		
24																								3	
25																									
26																									
